# Short-Term Effects of Land-Based Versus Water-Based Resistance Training Protocols on Post-Exercise Hypotension in Normotensive Men: A Crossover Study

**DOI:** 10.3390/sports10110181

**Published:** 2022-11-17

**Authors:** Gabriela Barreto David, Gustavo Zaccaria Schaun, Amanda Ricardo Mendes, Gabriela Neves Nunes, Danilo Sales Bocalini, Stephanie Santana Pinto, Cristine Lima Alberton

**Affiliations:** 1 Department of Sports, Physical Education School, Federal University of Pelotas, Pelotas 96055-630, Brazil; 2 Centre for Sport Science and University Sports, University of Vienna, 1150 Vienna, Austria; 3 Physiology and Biochemistry Laboratory, Physical Education and Sport Center, Federal University of Espírito Santo, Vitoria 29075-810, Brazil

**Keywords:** blood pressure, strength, aquatic exercises

## Abstract

Considering that water immersion may acutely reduce blood pressure (BP) and that exercise may elicit positive post-exercise hypotension (PEH) responses, we aimed to analyze the presence of PEH in normotensive individuals and compare its magnitude between two resistance training sessions performed in aquatic or land environments. Ten physically active men (23.2 ± 3.1 years) performed the two training protocols in a randomized, counterbalanced fashion. BP measurements were performed for 30 min (at 5 min intervals) both prior to (resting) and after each of the protocols. No differences were observed between protocols at baseline (*p* > 0.05). Only the water-based resistance training protocol resulted in a systolic BP reduction from 10 to 20 min post-exercise (all *p* < 0.05) compared to baseline. Compared to the land-based session, systolic BP was lower in the water-based protocol from 10 to 25 min post-exercise (all *p* < 0.05). On the other hand, diastolic BP showed a similar PEH effect between water and land-based protocols for the entire 30 min post-session period (all *p* < 0.001). Our results suggest that water-based resistance training holds the potential as a nonpharmacological strategy to lower BP levels following exercise.

## 1. Introduction

Hypertension is the most common risk factor for cardiovascular disease [1]. The number of adults with hypertension increased from 594 million in 1975 to 1.13 billion in 2015, with the increase seen primarily in low and middle-income countries. This increase is due mainly to a rise in hypertension risk factors in those populations, including insufficient physical exercise practice [2]. On the other hand, the 2020 World Health Organization (WHO) guidelines have reaffirmed moderate-certainty evidence from the effects of physical activity practice on health outcomes for adults, such as all-cause and cardiovascular disease [3]. Therefore, physical exercise is among the key nonpharmacological treatments for this condition [4] as an effective intervention to chronically reduce blood pressure levels. Similar to other exercise-related physiological adaptations, exercise effects on lowering blood pressure are associated with cumulative acute exposures to it [5]. These acute reductions observed in blood pressure levels after an exercise session to levels below those at rest, known as post-exercise hypotension (PEH), are thought to exert a substantial effect on the chronic adaptations of physical exercise in blood pressure management [6].

Water immersion is another intervention that may also affect blood pressure acutely [7,8]. During immersion, there is a rapid redistribution of blood toward the central region of the body, leading to an increase in cardiac output. Consequently, renal blood flow is increased, and a concomitant decrease in plasma renin activation and an increase in atrial natriuretic peptide concentrations are observed. Such alterations lead to an increase in diuresis and natriuresis associated with a reduction in both systolic (SBP) and diastolic (DBP) blood pressure levels [9]. Based on the premise that water immersion can reduce blood pressure acutely and that exercise may elicit positive PEH responses, it is plausible to suggest that water-based exercises could potentialize such responses.

Indeed, different water-based exercise protocols have been investigated to verify the presence of PEH in diverse populations such as older individuals [10,11,12], adults [13,14], and those with [15,16,17,18,19] or without [20] cardiometabolic disease. Moreover, in the last few years, some studies have observed the chronic effect of aquatic training on BP responses [21,22]. Ngomane et al. [17] found that SBP was reduced below resting levels 45 min after an aerobic training protocol performed in the water but not after a similar land-based protocol in older individuals with hypertension. Bocalini et al. [10] also observed a higher prevalence of PEH in normotensive and hypertensive patients 90 min after an aerobic training protocol performed in the aquatic compared to the land environment. Despite the positive results observed, the studies investigating PEH in the aquatic environment employed only aerobic or a combination of aerobic and resistance exercises. However, none of them investigated the PEH effects of an isolated resistance training protocol.

The 2020 WHO guidelines on physical activity and sedentary behavior describe a recommendation supported by moderate-certainty evidence regarding the additional health benefits on health outcomes through participation in muscle-strengthening activities at moderate or greater intensity on two or more days a week beyond aerobic exercises [3]. In addition, various forms of resistance training have been included as the top 20 Global Trends for 2022, according to the latest report by the American College of Sports Medicine [23], indicating their great popularity around the globe. Among the possible muscle-strengthening activities, water-based resistance training can be highlighted as an established training method for a wide range of individuals and, when adequately prescribed, can lead to significant gains in muscle strength [24].

Previous meta-analyses have shown that PEH effects can provide significant acute reductions in SBP and DBP after exercise. In addition, it may last up to 24 h after a land-based resistance exercise session in healthy and hypertensive individuals [2,5,25]. Nevertheless, to the best of our knowledge, no study to date sought to investigate the PEH responses to resistance training sessions performed in the aquatic environment. Although reductions in SBP and DBP are typically more pronounced in untreated hypertensive individuals compared to those who are treated and normotensive [10], the literature is still scarce regarding PEH in normotensive participants after water-based training protocols [14,20]. Therefore, more information is needed to verify the occurrence of PEH in this population.

Considering that the aquatic environment may end up conferring a cardioprotective effect through acute reductions in SBP and DBP during the exercise session compared to land, the possibility of a greater PEH response after water-based resistance exercise may prove advantageous when compared to conventional resistance exercise. To the best of our knowledge, scientific evidence is still insufficient; therefore, it is important to understand better the acute and chronic effects of water-resistance training to fill this gap. The purpose of the present study was to analyze the presence of PEH in normotensive individuals and compare its magnitude between water and land-based resistance training sessions. We hypothesized that both training protocols would result in PEH responses up to 30 min of recovery, with more pronounced effects in the aquatic environment.

## 2. Materials and Methods

### 2.1. Experimental Design

**Figure 1:**
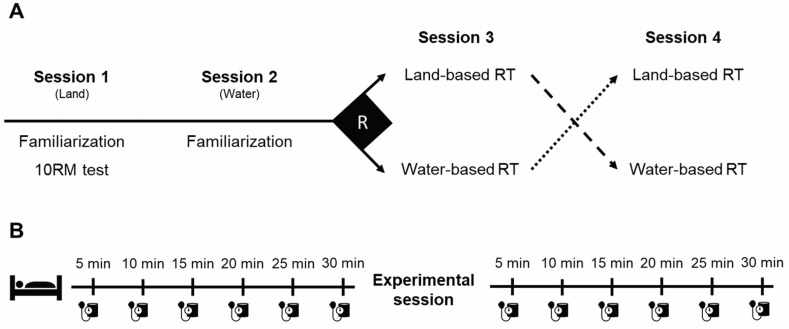
Experimental design. (**A**) After being familiarized with both land and water-based exercises, participants were randomly assigned to perform one of the resistance training protocols (RT) and, at least 48 h later, the remaining one. (**B**) Blood pressure outcomes were assessed for 30 min before and after exercise in each experimental session (i.e., sessions 3 and 4).

To fulfill the purpose of the present investigation, participants attended four experimental sessions (Figure 1). In the first session, they were familiarized with the resistance exercises and performed 10 repetitions maximum (10 RM) tests to determine the land-based resistance training exercise loads. The second session participants were familiarized with the water-based resistance training protocol. The remaining two sessions corresponded to the protocols for blood pressure data assessment (2 × 2 crossover study), whose order was randomly defined. These sessions were separated by at least 48 h. The protocol order was assigned to the first participant by simple randomization, and the following participants were assigned in a counterbalanced order. Throughout the study, all volunteers were instructed to avoid intense physical activity within 24 h before the experimental protocols.

### 2.2. Participants

Ten young male university students volunteered to participate in the present study. To be eligible, participants had to be 18 to 30 years old, normotensive, and currently engaged in physical activity at least twice a week (e.g., aerobic and/or resistance exercises or individual/team sports) but not be athletes. At the time of enrollment, they also had to be free of any known musculoskeletal, bone, joint, cardiac, or pulmonary disease. They should not have taken any medication that could interfere with the investigated outcomes. In addition, participants had to be familiarized with the aquatic environment to provide more reliable measures.

The sample size calculation was performed using the GPower v. 3.1 software, adopting a significance level of α = 0.05, a power of 95%, and an effect size f of 0.65. Data for sample size calculation were extracted from Rodriguez et al. [14] using HPE for SBP and DBP outcomes after water versus land-based exercise sessions in healthy individuals, resulting in a total sample size of 8 participants. Two additional participants (i.e., ~20%) were recruited to account for possible losses. Before any procedure, all participants were carefully informed about the study design and the potential risks and discomforts related to the study’s procedures and signed informed consent. The study was approved by the Local Ethics Committee (CAAE: 51771415.4.0000.5313) and was developed following the Declaration of Helsinki.

### 2.3. Procedures

#### 2.3.1. Familiarization

*Familiarization with land-based resistance exercises*. Participants attended the first experimental session in which they were assessed for characterization data and familiarized with the exercises of the land-based resistance training protocol. First, body mass and height were measured using a digital scale (Welmy, Santa Barbara d’Oeste, Brazil) and a manual stadiometer. Skinfold measures were obtained using a skinfold caliper (Cescorf, Porto Alegre, Brazil) to estimate body density [26], and Siri’s equation [27] was used to calculate body composition.

Participants warmed-up for 5 min in a cycle ergometer and experienced all land-based resistance exercises used during the protocol, with particular attention given to the technique and range of motion. Following their familiarization, all participants were tested for their 10 RM loads, used for determining the land-based resistance training protocol intensity. For this purpose, an expert investigator determined an initial load to start each 10 RM test based on the previous familiarization. Participants were then asked to perform the maximal number of repetitions with the assigned load. The repetition rhythm was controlled (2 s for each phase), and the workload was adjusted after each attempt according to Lombardi’s coefficients as necessary [28]. Each exercise test consisted of five attempts with 3 min rest intervals between sets and exercises. The load corresponding to 10 RM was chosen because it is within the range widely used in land-resistance training programs. In addition, its corresponding duration is within the range commonly used in water-resistance training programs.

*Familiarization with water-based resistance exercises.* Participants were fully familiarized with the aquatic environment and the water-based resistance exercises used during the corresponding protocol in a separate session. In addition, participants were also trained with their perceived maximal effort and instructed to perform each repetition at maximal velocity and range of motion, which is how resistance exercises are typically prescribed in the aquatic environment for young individuals [29,30].

#### 2.3.2. Resistance Training Protocols

Both land and water-based training protocols were designed to match total duration, muscle group involvement, and time of intervals. The load intensity and muscle recruitment pattern differed in each environment, considering their specificities.

**Figure 2:**
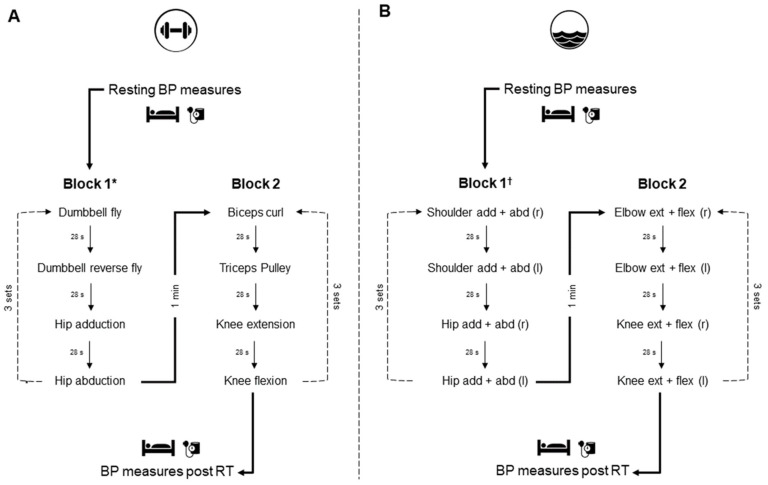
Land-based (**A**) and water-based (**B**) resistance exercise protocols. Resting BP outcomes were measured for 30 min before and after participants completed the protocols, which comprised two blocks of exercises performed in a circuit manner. Each block was performed three times with 28 s recovery periods between exercises and 1 min between blocks. * = Land-based resistance exercises were performed for a total of 8 repetitions per set; ^†^ = Water-based resistance exercises were performed for a total of 32 s at maximum velocity per set; Both protocols lasted 25 min. r = right limb; l = left limb; add = adduction; abd = abduction; ext = extension; flex = flexion.

*Land-based training protocol.* Land-based training protocol. As shown in Figure 2, the land-based resistance training protocol was composed of two blocks of four exercises performed in a circuit manner. Three sets of eight repetitions were performed for each exercise at the load corresponding to the 10 RM test (i.e., submaximal intensity to avoid concentric failure). Exercises were performed bilaterally, with both concentric and eccentric movement phases controlled at 2 s, lasting a total of 32 s per set (i.e., 8 reps × 4 s = 32 s). A 28 s passive recovery between sets and exercises was provided (12 min per block). In addition, an interval of 1 min was considered between blocks for a total protocol duration of 25 min. The current land-based resistance training protocol was designed to match its period with the water-based resistance training protocol. Water-based resistance exercises are typically prescribed based on time intervals and not on a range or pre-determined number of repetitions [29,30].

*Water-based training protocol.* As shown in Figure 2, the water-based resistance training protocol was composed of two blocks of two exercises performed unilaterally in a circuit manner. Three sets of 32 s of effort were performed for each exercise at maximum velocity and range of motion. A 28 s passive recovery between sets and exercises was provided for participants to get in position for the following exercise (12 min per block). In addition, an interval of 1 min was considered between blocks for a total protocol duration of 25 min.

The water-based resistance training protocol structure was based on the notion that, different from what typically happens in traditional resistance training, in the aquatic environment, each exercise phase recruits both agonist and antagonist muscle groups with concentric and eccentric contractions due to water resistance [31]. This protocol was adapted from the protocol proposed by Schoenell et al. [30], which demonstrated positive adaptations in muscle strength in young individuals after 10 weeks of training. Immersion depth was maintained between the xiphoid process and shoulders level, and the water temperature was kept between 31 and 33 °C.

### 2.4. Outcome Assessments

Blood pressure was assessed with an automated oscillometric device (7200; OMRON, São Paulo, Brazil) positioned on the participants’ right arm. Specifically, with participants lying in a supine position in a silent and calm room, SBP and DBP were measured at 5 min intervals for 30 min before and after the completion of the land-based and water-based resistance exercise protocols (as detailed in Figure 1 and Figure 2). These measures were always conducted by the same investigator, who was not blind to the intervention’s nature. Both SBP and DBP were initially compared at rest within (at each 5 min time point) and between protocols. The 20 min resting time point was selected as the baseline, as used by Pinto et al. [20], to investigate the PEH effect of these protocols and compared to the post-exercise blood pressure results at the 5th, 10th, 15th, 20th, 25th, and 30th min time points, both within and between protocols.

### 2.5. Statistical Analyses

Descriptive data are reported as mean ± standard deviation (SD) or 95% confidence interval (CI). Normality and sphericity were tested using Shapiro–Wilk and Mauchly tests, respectively. Two-way repeated measures ANOVAs were used to compare SBP and DBP values between land and water-based resistance protocols and time points. When an interaction effect was significant, post hoc comparisons were performed using the Bonferroni test for each main effect. The between-protocol effect size of the PEH magnitude for each time point was calculated using Cohen’s d and considered as very small (<0.1), small (0.1–0.2), medium (0.2–0.5), large (0.5–0.8), very large (0.8–1.2), and huge (2.0) as described by Sawilowsky [32]. The significance level was set to α = 0.05, and all statistical tests were performed in the SPSS statistical software package (version 20.0 for Windows; SPSS Inc., Chicago, IL, USA).

## 3. Results

**Figure 3:**
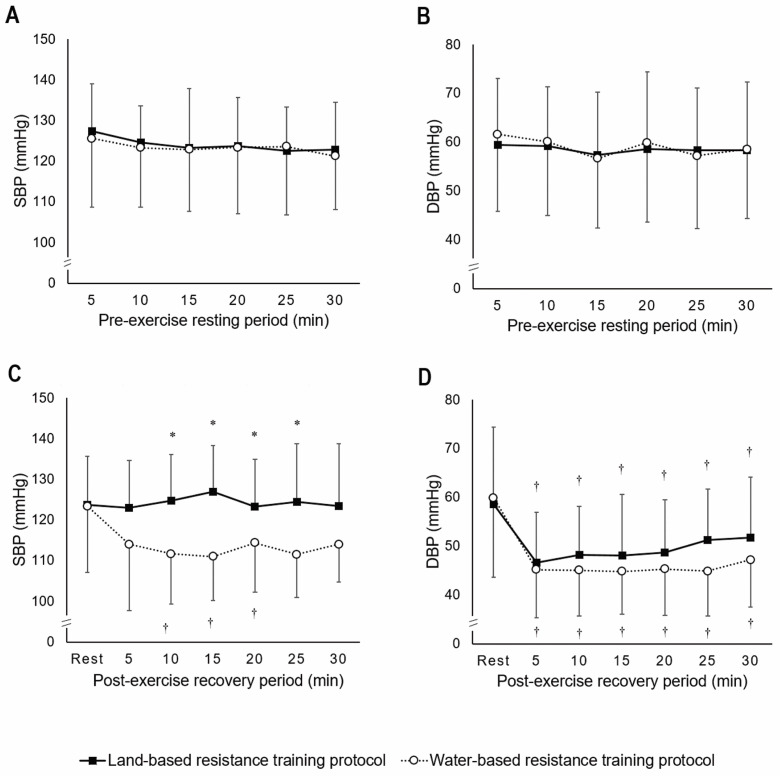
Resting systolic (SBP) (**A**) and diastolic (DBP) (**B**) blood pressures between land and water-based resistance training protocols. SBP (**C**) and DBP (**D**) at rest and throughout 30 min post-exercise between water-based and land-based resistance protocols. * Significantly different from the water-based resistance training session; ^†^ Significantly different from the rest time point (*p* < 0.05).

Participants completed both exercise sessions, and no adverse events were observed. Descriptive data of the sample characteristics are presented in Table 1. At rest, both SBP and DBP values remained stable throughout the entire 30 min period, and no differences were observed between the protocols. These results confirm that participants started both exercise training sessions from the same basal condition (Figure 3A,B).

As for the post-exercise comparisons, a significant protocol*time point interaction was observed for SBP (F_(6;54)_ = 2.596; *p* = 0.028), indicating a different blood pressure behavior after exercise between the resistance training protocols investigated (Figure 3C). In the land-based protocol, no significant PEH was observed for SBP (F_(6;54)_ = 0.599; *p* = 0.730), whereas a significant PEH was observed in the water-based resistance protocol from 10 to 20 min after the session in comparison to the resting period (F_(2.7;23.9)_ = 3.014; *p* = 0.05). Water-based resistance training resulted in lower SBP values from 10 to 25 min post-exercise compared to the land-based protocol.

Contrary to what was observed in the SBP values, no significant protocol*time point interaction was found for DBP (F_(2.4;21.2)_ = 1.328; *p* = 0.289), therefore indicating a similar post-exercise DBP behavior in both the protocols investigated (Figure 3D). Accordingly, a significant diastolic PEH was observed at all time points (from 5 to 30 min) in comparison to the baseline (F_(2.6;23.5)_ = 16.835; *p* < 0.001), with no significant difference between water and land-based resistance training protocols (F_(1;9)_ = 3.014; *p* = 0.117).

A descriptive summary of systolic and diastolic PEH absolute deltas through the entire 30 min post-exercise period related to the resting period is also available and presented in Table 2.

## 4. Discussion

The present study’s main finding was that only the water-based resistance protocol induced a significant PEH on SBP (from 10 to 25 min post-exercise) in normotensive young men. In contrast, both water and land-based protocols resulted in a short-term PEH effect on DBP (i.e., from 5 to 30 min post-exercise).

In line with our findings, a PEH effect after resistance exercises has been clearly demonstrated in hypertensive individuals [33,34,35] and often in healthy normotensive adults as well [36,37,38], although to a lower magnitude [2,5,25,39]. Keese et al. [37] observed an approximately 2 mmHg reduction in DBP 20 min after a land-based resistance protocol (eight exercises, 3 × 80% 1 RM) in healthy young men, compared to pre-session. Similarly, a land-based resistance protocol (six exercises, 3 × 65% 1 RM) also resulted in an approximately 9 mmHg DBP reduction in trained men up to 30 min post-exercise in comparison to pre-session [36], which is within the −7 to −13 mmHg range observed in our study for the land-based protocol. Nevertheless, these studies verified lower SBP levels (−4 and −10 mmHg, respectively) after exercise. In this regard, the literature agrees that a PEH effect occurs after resistance exercise sessions. However, it is relatively inconsistent on how PEH is manifested, especially in normotensive individuals, with studies showing a reduction after land-based resistance protocols only in the SBP, in the DBP, or in both SBP and DBP [2,5,25].

With respect to water-based protocols, studies regarding the effects of aerobic and combined (aerobic and resistance) exercise sessions also consistently report a PEH in hypertensive [10,11,13,16,17,18] and healthy normotensive individuals [14,20]. Pinto et al. [20] showed a short-term diastolic PEH 10 min after the session in normotensive young women after a session of combined resistance (4 exercises, 20 s of stimulus at maximal velocity) and aerobic (heart rate corresponding to the anaerobic threshold) water-based protocol (−13 mmHg). In contrast, no significant PEH was observed in the session with inverse order (i.e., aerobic and resistance exercises). Caution should be taken in this analysis because the literature has shown different acute and chronic physiological responses between isolated aerobic and isolated resistance water-based sessions or programs [40,41]. Nevertheless, to the best of our knowledge, the present study is the first to investigate the PEH effects of an isolated resistance training protocol performed in the aquatic environment.

Regarding environment comparison, our results demonstrate a PEH effect on SBP and DBP in normotensive young men after the water-based resistance protocol, while the protocol performed on land only resulted in diastolic PEH, which is partially in line with our initial hypothesis. Previous studies analyzing the environment’s effect on PEH investigated only aerobic and combined protocols [10,12,14,18]. They observed HPE for SBP and DBP after both training modes, often to a greater magnitude in water-based protocols. In normotensive older individuals, Bocalini et al. [10] compared the short-term PEH responses between water and land-based aerobic protocols (45 min 75% VO_2max_). The authors verified a greater SBP reduction 30 to 90 min post-exercise in the water than on land (−10 mmHg vs. −4 mmHg). On the other hand, DBP was reduced 30 and 45 min post-exercise only in the water-based protocol (≈−4 mmHg).

To date, we are only aware of one investigation that compared the PEH effect between water and land environments in trained and untrained healthy normotensive adults [14]. Using a walking protocol (40% VO_2max_), the authors reported that, in the untrained women, the PEH for SBP (−9 to −11 mmHg) and DBP (−5 to −7 mmHg) was evident after the water-based aerobic protocol only, whereas no PEH was observed after land-based protocol. Conversely, the trained women presented PEH for SBP in both environments (−9 to −11 mmHg and −8 to −10, respectively), while PEH was observed in DBP only 60 min of land-based protocol (−4 mmHg). The magnitude of the SBP and DBP reductions observed in our water-based resistance protocol (−9 to −12 mmHg and −11 to −16 mmHg, respectively) are similar to or greater than those previously reported in water-based aerobic and combined protocols [12,14,18]. The DBP reductions are even comparable to those in hypertensive individuals [12,18]. Collectively, these results reinforce the potential of water-based resistance training protocols to acutely reduce blood pressure after exercise, even when compared to a protocol performed on land involving matched total muscle mass involved and total duration.

In this regard, the acute reductions in blood pressure after exercise observed in the present investigation may be explained by reductions in the peripheral vascular resistance (i.e., increased systemic vascular conductance) through mechanisms such as reduced sympathetic activation and transduction of sympathetic outflow and also by local release of vasodilating factors, such as histamine [5,42]. The PEH for SBP present in the water-based resistance protocol but not in the land-based one might be related to different possibilities. First, water immersion results in a hydrostatic pressure-driven compression of the superficial blood vessels that facilitates venous return, leading to greater atrial stretching, especially in the right atrium [43]. Consequently, there is a stimulation of low-pressure cardiac baroreceptors that may inhibit sympathetic nerve activity. Secondly, the lower sympathetic activation and the increased blood flow to organs such as the kidneys lead to several acute hormonal changes. Reductions in the antidiuretic hormone, greater atrial natriuretic peptide release, renin–angiotensin system suppression, and hypothalamic–pituitary–adrenal axis suppression result in lower cortisol and catecholamines secretion [9,44,45,46]. As a result, diuresis is increased [43] to recover basal plasma volume.

In addition, by comparing work-matched concentric and eccentric resistance exercises, Stavres et al. [38] observed that the former augmented PEH compared to eccentric contractions. As such, at least part of the differences observed in SBP between both training protocols may be related to the distinct contraction profiles found in the aquatic and land environments. Specifically, traditional resistance exercises performed on land are typically characterized by marked concentric and eccentric contraction of the same agonist musculature. In contrast, water-based resistance exercises are characterized as cycles of agonist concentric contraction to accelerate the movement, followed by antagonist eccentric contraction to decelerate the movement [30]. Therefore, the eccentric component in water-based resistance exercises probably occurs at a lower magnitude when compared to traditional resistance exercises performed on land. Furthermore, on land, the specific tension occurs in the fibers of the same agonist muscle group during both concentric and eccentric actions, while in water, the specific tension in the eccentric action occurs in the fibers of a distinct antagonist muscle group, resulting in lower levels of muscle damage [47].

Finally, concerning the chronic effects, a recent study [48] compared the efficacy of five types of exercise programs (continuous endurance training, interval training, resistance training, combined training, and hybrid-type training) on cardiometabolic health parameters among adults with normal blood pressure levels. Their network meta-analysis ranked combined training as the best exercise treatment for lowering SBP and DBP. These results reinforce the importance of including resistance exercise in addition to aerobic one during programs that aim to improve cardiometabolic health, regardless of the environment, evidencing the novelty of present findings for the aquatic environment.

The study’s main strengths were the original attempt to compare acute blood pressure responses in resistance training programs performed in different environments by matching some parameters of session prescription in a crossover study design. On the other hand, some limitations should be addressed in the present study. First, the present study has no control session. Nevertheless, we performed a 30 min control period before the exercise sessions, and no differences were observed in resting blood pressure levels between the water and land-based resistance exercise sessions, indicating that they started from similar baseline levels. Furthermore, a control session in the water would benefit our design since it would provide a better understanding and differentiate the PEH effects related to immersion or the water-based resistance protocol separately. Another possible limitation is the difficulty of controlling the training workload in the water environment and, consequently, equalizing loads between both protocols. It is important to note, however, that both protocols were matched for total duration and total muscle mass involved, both of which can influence PEH responses [2,25]. Finally, the participants’ hydration and plasma volume levels were not controlled. Their composition may influence cardiovascular system adjustments, and autonomic modulation may have influenced blood pressure during and after both resistance exercise protocols due to its impact on diuresis and promote some influence on arterial stiffness. Future studies are required where the SBP and DBP changes following both water-based and land-based resistance training protocols are monitored using ambulatory monitoring for up to 24 h. Moreover, HPE for similar resistance training protocols should be investigated in hypertensive patients.

## 5. Conclusions

In conclusion, water-based resistance exercise resulted in a significant PEH effect for SBP and DBP in young normotensive individuals, whereas only DBP was reduced after a similar land-based protocol. More importantly, SBP reductions were greater 10 to 25 min after the water-based protocol than the land-based one. Resistance training interventions typically show mean SBP and DBP reductions ranging from 2 to 4 mmHg, close to those observed with antihypertensive drug administration [25,49]. Considering that PEH responses are typically greater in pre-hypertensive and hypertensive individuals [2,25,50], the present PEH results observed in healthy normotensive men suggest that water-based resistance training holds potential as a nonpharmacological strategy to lower blood pressure levels following exercise and assist on the blood pressure maintenance in normotensive individuals. Therefore, the findings reinforce that the aquatic environment potentializes the PHE responses. Water immersion per se is an intervention that may also affect blood pressure acutely, reducing it at rest or during exercise. Based on this, resistance training sessions performed in the aquatic environment may be advantageous for cardiometabolic health parameters compared to traditional resistance exercises on land.

## Figures and Tables

**Table 1:** Descriptive data of sample characterization.

Variables	Mean ± SD
Age (years)	23.2 ± 3.12
Body mass (kg)	75.7 ± 9.0
Height (cm)	175.0 ± 0.3
Body fat (%)	13.5 ± 3.0

**Table 2:** Mean, 95% confidence interval (CI), and between-protocol effect size (d) of the magnitude of the post-exercise hypotension in systolic (SBP) and diastolic (DBP) blood pressure levels after water-based and land-based resistance protocols.

Variables	Land-Based Resistance Training Protocol	Water-Based Resistance Training Protocol	
	Mean (95% CI)	Mean (95% CI)	*d* (95% CI)
*SBP (mmHg)*			
5 min post	−4.4 (−10.2 to 1.4)	−11.6 (−20.9 to −2.3)	0.67 (−0.24 to 1.47)
10 min post	0.1 (−6.5 to 6.7)	−11.6 (−21.1 to −2.1)	1.02 (0.06 to 1.83)
15 min post	3.7 (−3.9 to 11.3)	−11.9 (−21.2 to −2.6)	1.32 (0.30 to 2.12)
20 min post	−0.5 (−7.2 to 6.2)	−9.0 (−17.8 to −0.2)	0.78 (−0.14 to 1.59)
25 min post	2.0 (−3.9 to 7.9)	−12.1 (−23.1 to −1.1)	1.00 (0.04 to 1.80)
30 min post	0.5 (−7.0 to 8.0)	−7.3 (−14.6 to 0.0)	0.75 (−0.17 to 1.56)
*DBP (mmHg)*			
5 min post	−12.8 (−17.8 to −7.8)	−16.4 (−23.2 to −9.1)	0.41 (−0.46 to 1.22)
10 min post	−11.0 (−16.7 to −5.3)	−15.0 (−24.0 to −6.0)	0.38 (−0.49 to 1.19)
15 min post	−9.3 (−14.9 to −3.7)	−11.9 (−19.9 to −3.9)	0.27 (−0.59 to 1.09)
20 min post	−9.9 (−16.1 to −3.8)	−14.6 (−21.7 to −7.5)	0.50 (−0.38 to 1.31)
25 min post	−7.0 (−13.1 to −0.9)	−12.3 (−19.5 to −5.1)	0.52 (−0.37 to 1.33)
30 min post	−6.7 (−13.3 to −0.9)	−11.4 (−17.5 to −5.3)	0.53 (−0.36 to 1.34)

## Data Availability

Data relating to this article will be made available upon request to the corresponding author.
